# Agreement between retrospective and prospective assessments of childhood abuse revisited

**DOI:** 10.1017/S0954579424001032

**Published:** 2024-06-04

**Authors:** Marissa D. Nivison, Clarissa R. Filetti, Elizabeth A. Carlson, Deborah B. Jacobvitz, Glenn I. Roisman

**Affiliations:** 1 Institute of Child Development, University of Minnesota Twin Cities, Minneapolis, MN, USA; 2 Human Development and Family Sciences, University of Texas at Austin, Austin, TX, USA

**Keywords:** Adult Attachment Interview, child abuse, early caregiving, longitudinal, prospective, retrospective

## Abstract

A recent meta-analytic review demonstrated that retrospective assessments of childhood abuse acquired during adulthood – typically via self-report – demonstrate weak agreement with assessments of maltreatment gathered prospectively. The current report builds on prior findings by investigating the agreement of prospectively documented abuse from birth to age 17.5 years in the Minnesota Longitudinal Study of Risk and Adaptation with retrospective, Adult Attachment Interview-based assessments of childhood abuse administered at ages 19 and 26 years. In this sample, an agreement between prospective and retrospective assessments of childhood abuse was considerably stronger (*κ* = .56) than was observed meta-analytically. Retrospective assessments identified prospectively documented sexual abuse somewhat better than physical abuse, and the retrospective approach taken here was more sensitive to identifying abuse perpetrated by primary caregivers compared to non-caregivers based on prospective records.

Childhood maltreatment has been demonstrated to have detrimental, cascading effects on human development across the life span. Indeed, research investigating childhood abuse has arguably reached its apotheosis over the past 25 years by way of the development and widespread use of various measures of adverse childhood experiences (ACEs; Felitti et al., 1998). Such self-reported, retrospective measures about difficult early experiences are routinely administered to adults in a variety of high-stakes contexts, including healthcare settings and in the educational sector, in addition to serving as a primary method in much of the basic and translational research now being conducted on maltreatment. For example, a recent meta-analysis focused on childhood maltreatment found that only about 33% of existing studies assess abuse prospectively (Li et al., 2016).

The various ACEs measures have clear value both because they (a) provide a direct assessment of adults’ subjective appraisals of their early life experiences and (b) generally involve a simple binary checklist of adverse childhood events (e.g., physical and sexual abuse) that is easily administered and scored. In turn, such tallies of adverse experiences have been shown to be robustly associated with a variety of (negative) developmental outcomes, including alcoholism, depression, poor self-rated health, and diagnosis of serious adult illnesses (Felitti et al., 1998). That said, the significance of such findings hinges critically on whether it is possible to measure early caregiving experiences validly in a retrospective manner, a methodological issue that has bedeviled developmental science for almost 90 years (e.g., Nivison et al., 2021a; Pyles et al., 1935; Yarrow et al., 1970).

One aspect of validity in this context is to what extent retrospective and prospective reports of childhood maltreatment show nontrivial agreement (e.g., Newbury et al., 2018; Reuben et al., 2016). Reuben et al. (2016), for example, found that retrospective and prospective measures of ACEs converged only moderately (*κ* = .31) in the Dunedin Multidisciplinary Health and Development Study. Additional evidence from the Environment Risk Longitudinal Twin Study similarly showed weak agreement between retrospective and prospective measures of maltreatment (*κ* = .19), though when subdivided by maltreatment type, agreement was somewhat higher for sexual abuse (*κ* = .31) compared to physical abuse (*κ* = .20; Newbury et al., 2018). These authors concluded that retrospective and prospective measures of ACEs capture largely nonoverlapping groups of individuals.

A recent meta-analysis of all relevant evidence reported findings consistent with the results of the landmark longitudinal investigations noted immediately above. More specifically, Baldwin et al. (2019) meta-analyzed data from sixteen studies and found that retrospective and prospective measures of childhood maltreatment had poor agreement (*κ* = .19), though convergence was somewhat stronger when retrospective measures were interview-based (*κ* = .22) rather than self-reports (*κ* = .11). Current scientific consensus is that retrospective and prospective measures of childhood abuse capture different groups of people and cannot be used interchangeably. However, one reason why existing prospective and retrospective measures of maltreatment might fail to agree is that most retrospective assessments of maltreatment focus on adults’ subjective experiences of childhood abuse. That is, retrospective measures of abuse tend to be both retrospective and subjective in nature. Although such measures have utility in that they provide insights into individuals’ perceptions of their own early experiences (Barnett et al., 1993; Danese, 2020), our goal here was to determine whether at least some of the disagreement between prospective and retrospective approaches to the assessment of childhood abuse might be eliminated via leveraging interview-based methods, consistent with meta-analytic evidence (Baldwin et al., 2019).

Of note, interview-based methods for assessing childhood maltreatment already exist. However, many such measures are often self-reports that have been merely restructured as interviews (see Widom & Shepard, 1996). This point is important to emphasize because such measures still require that individuals explicitly construe their experiences as abuse. Doing so can be challenging for some adults, in part because the way individuals discuss their childhood experiences is not always consistent with the explicit information they report, a phenomenon that has been well documented in the adult attachment literature (e.g., Roisman, 2009). For example, an adult might report that their childhood relationship with their caregiver was loving, but when asked to provide specific memories to support such a characterization, generate a narrative that is in direct opposition to the general descriptors they offer to describe their early experiences and thereby indirectly provide evidence of harsh parenting and even abuse.

In the current study, therefore, we leverage an existing, semi-structured protocol focused on adults’ childhood experiences with primary caregivers (including experiences with childhood abuse) known as the Adult Attachment Interview (AAI). More specifically, we recoded AAIs from the Minnesota Longitudinal Study of Risk and Adaptation (MLSRA; Sroufe et al., 2005) using a scale that was specifically developed to identify experiences of childhood physical and sexual abuse retrospectively (Jacobvitz et al., 2006; Leon et al., 2004). In contrast to most retrospective measures, however, this coding scale developed by Jacobvitz et al. (2006) does not rely on adult participants to construe their experiences as abuse and thus has the potential to agree to a greater extent with prospective assessments of childhood maltreatment than other existing retrospective measures.

## The present study

The present study draws on the MLSRA (Sroufe et al., 2005). The MLSRA is a prospective, longitudinal study that has followed participants from three months prior to the child’s birth through midlife. The MLSRA contains extensive, prospectively documented childhood abuse and neglect data from birth to 17.5 years of age. Abuse and neglect data were generated through multiple methods and by leveraging multiple informants. Later, at ages 19 and 26 years of age, participants were administered the AAI. Although the AAI was not designed to serve as a retrospective assessment of maltreatment, the interview does probe for childhood abuse and specifically asks interviewees if they experienced childhood physical and sexual abuse by any perpetrator (i.e., not just primary caregivers). For the present report, the age 19 and 26-year AAIs were recoded for evidence of abuse using an existing coding system noted earlier (i.e., Leon et al., 2004). This retrospective coding system focuses on experiences of harsh physical punishment such as repeated hitting as well as experiences of sexual abuse.

As already noted, the present study examined agreement between prospectively assessed abuse from birth to age 17.5 years and an overall measure of retrospectively reported abuse at ages 19 and 26 years. Given the prospective data available in the MLSRA, we were also well positioned to examine specific parameters of abuse, including subtype (i.e., physical vs. sexual abuse) and perpetrator (i.e., mother figure, father figure, or non-caregiver). Although these more nuanced analyses were largely exploratory, we concluded that it was important to examine agreement beyond a simple dichotomous variable (presence vs. absence of abuse) given calls from within the maltreatment literature for more nuanced parameterization of maltreatment in relevant research (e.g., Cicchetti, 2013). As a result, the current study addressed two aims. First, we examined to what extent retrospectively recalled abuse in young adulthood overlaps empirically with prospectively documented abuse from birth to 17.5 years (Aim 1). Second, we determined to what extent the agreement of retrospective and prospective measures of abuse varies by specific parameterizations of prospectively documented abuse (i.e., type [Aim 2a] and perpetrator [Aim 2b]).

It is important to note that the 19-year AAIs (but not the 26-year AAIs) in the MLSRA have been previously coded for retrospective abuse (Shaffer et al., 2008). However, these investigators used a different coding system that had been developed based on the criteria outlined by Barnett et al. (1993). Although the Barnett et al. (1993), coding scheme is rigorous and detailed, it was not designed specifically for use in the AAI. Therefore, instead of using the existing data and coding system, for this study, we recoded both the 19- and 26-year AAIs using a system that was developed specifically for the AAI (i.e., Jacobvitz et al., 2006; Leon et al., 2004).

## Methods

### Participants

Participants were drawn from the MLSRA (Sroufe et al., 2005), a prospective longitudinal study following mothers and their children from 3 months before the child’s birth to over 40 years of age. The child was the target participant of the MLSRA, but mothers (as well as grandparents, teachers, etc.) were often assessed as it pertained to the development of the child (e.g., parent-child interactions, maternal depressive symptoms). Between 1975 and 1977, 267 pregnant mothers seeking free prenatal care in Minneapolis, Minnesota, were recruited to participate. Consistent with the goals of this study at onset, which included the study of children at risk for maltreatment, expectant women were recruited if they were living at or below the poverty line prior to their child’s birth. Forty-eight percent of participants were adolescents, 65% were single, and 42% had not completed a high school education. The present analyses focused on participants who completed at least one codable AAI at either 19 or 26 years of age and who also have complete prospective abuse data. The subsample was thus comprised of 162 participants (48% female, 68% White/non-Hispanic) overall, though the sample size varies by parameterization of abuse. The subsample (*N* = 162) did not differ from the original sample (*N* = 267) on socioeconomic status, ethnicity/race, or sex assigned at birth. However, the present subsample (*n* = 162, *M* = 12.30, *SD* = 1.59) when compared to those excluded (*n* = 104, *M* = 11.73, *SD* = 2.01) had significantly higher maternal education (*t* [183.45] = −2.41, *p* = .02, equal variances not assumed). Despite this, average levels of maternal education in the current sample were still equal to or less than a high school education, consistent with an at-risk cohort. The follow-up of MLSRA and related analyses were approved by the University of Minnesota ethics review board (title: Early Life Stress, Developmental Processes, and Adult Health, IRB ID 1104S98312).

### Measures

#### Prospective abuse

The MLSRA assessed a variety of atypical parent-child experiences that were prospectively measured. These included participants’ adverse caregiving experiences of physical abuse, sexual abuse, and neglect. Several years ago, this information was recoded and standardized to apply contemporaneous definitions of abuse and neglect, to identify the specific perpetrator and ages of the abuse and neglect experiences, and to assess the reliability of those coding decisions. Coding criteria were initially based on definitions developed by the Centers for Disease Control and Prevention (CDC) in order to “promote consistent terminology and data collection related to child maltreatment” (Leeb et al., 2008, p. 4). The coding included (a) neglect of a child’s basic physical or cognitive needs, defined as a caregiver’s failure to provide adequate hygiene, shelter, clothing, medical care, supervision, or education; (b) physical abuse, defined as a caregiver’s “intentional use of physical force against a child that results, or has the potential to result in, physical injury” (Leeb et al., 2008, p. 14); and (c) sexual abuse, defined as sexual contact (e.g., molestation, rape) or noncontact exploitation (e.g., intentional exposure of the child to pornography) by a custodial caregiver or by a perpetrator 5 or more years older than the target child. Although the CDC criteria only address sexual abuse perpetrated by a caregiver, the inclusion of non-caregiving perpetrators and the use of a 5-year cutoff is consistent with other research in this area (e.g., Stoltenborgh et al., 2011).

Additionally, in order to be consistent with the literature and Minnesota state guidelines, the CDC definitions were supplemented by a set of more specific coding guidelines that distinguished clear indicators of physical abuse, sexual abuse, and physical/cognitive neglect from ambiguous indicators that were not sufficient for classification in isolation of other evidence. These additional guidelines were developed in consultation with MLSRA senior researchers, Minnesota state law, and available research literature (e.g., Barnett et al., 1993) and are available from the first author upon request. However, the classifications of childhood experiences of abuse or neglect do not necessarily reflect the criteria for maltreatment used by child protective services, which vary from state to state. As such, our scoring of abuse and neglect does not necessarily mean that these children or their families were involved with child protective services.

Although emotional unavailability or lack of caregiver responsive- ness has proven to be an important dimension of adverse caregiving (especially for young children), with pernicious developmental consequences (National Scientific Council on the Developing Child, 2012; Sroufe et al., 2005), this dimension was not included in the current coding criteria due to insufficient information across developmental periods. Similarly, exposure to violence between caregivers and other forms of environmental violence were not included in the current set of codes. Exposure to violence between caregivers is captured by a separate variable in the MLSRA data set (e.g., Narayan et al., 2013), and insufficient information was available to code adequately exposure to other forms of environmental violence.

In the MLSRA, 139 participants (52% of the original sample) had been previously flagged as potentially having experienced abuse and/or neglect. Therefore, for these cases, all available data collected from birth to 17.5 years (up to 25 assessments) were reviewed for more detailed information regarding caregiving quality, physical discipline, supervision, home environment, physical and sexual assault, child protective service involvement, and foster care history in order to make judgments about whether abuse had occurred. Information was obtained from parent-child observations, caregiver interviews, reviews of available child protection and medical records, adolescent reports, and teacher interviews. Disclosure of physical or sexual abuse during the AAI (George et al., 1985) was consulted for the prospective abuse coding only if an experience of abuse was initially identified based on records through age 17.5 years, but there was insufficient detail to code the specific developmental period or perpetrator. The AAI only provided a means to clarifying existing knowledge gleaned from prospective sources. Therefore, the present analyses are not conflated by shared measures for both the retrospective and prospective coding of abusive experiences.

Coding focused on the presence or absence of physical abuse, sexual abuse, and/or neglect in each of four developmental periods (infancy: birth to 24 months; early childhood: 25 months to 5 years; middle childhood: 6–12 years; and adolescence: 13–17.5 years). For incidents of physical and sexual abuse, coders additionally specified the perpetrator. Perpetrators included maternal caregivers (biological mothers, stepmothers, grandmothers), paternal or father figures (biological fathers, stepfathers, adoptive fathers, and mothers’ live-in boyfriends), and non-parental figures (relatives, neighbors, babysitters, and family friends). Two coders reviewed each case and demonstrated good to excellent reliability for all parameters: kappas were between .80 and .98 for the presence or absence of physical abuse, sexual abuse, and/or neglect, .80 and .84 for the presence or absence of each type during each development period, and .80 and .98 for incidents of physical or sexual abuse by each category of perpetrator. All discrepancies were resolved by consensus.

Within the full sample of MLSRA participants (*N* = 267), 102 individuals were classified as having ever experienced physical abuse, sexual abuse, and/or neglect; 81 were coded as not having experienced abuse or neglect; and the status of 84 was deemed unclear due to missing data (see below). By developmental period, 47 individuals were classified as being abused and/or neglected in infancy (of the 211 with sufficient data to allow for confident classifications of abuse and/or neglect during this developmental period), 66 in early childhood (of the 185 with sufficient data during this developmental period), 66 in middle childhood (of the 190 with sufficient data during this developmental period), and 21 in adolescence (of the 179 with sufficient data during this developmental period).

Within the current sample of participants who had at least one AAI in young adulthood (*N* = 178), 69 individuals were classified as having ever experienced physical or sexual abuse. Among participants with histories of abuse, 46% had experienced sexual abuse, and 74% had experienced physical abuse (not mutually exclusive). Within the abused group, 14% experienced abuse in infancy, 46% during early childhood, 65% during middle child- hood, and 28% during adolescence (not mutually exclusive). In terms of chronicity, 54% of this group experienced abuse during one developmental period, 25% during two periods, 12% during three periods, and 1% during all four developmental periods; 8% had insufficient data to determine the number of developmental periods during which abuse occurred. Among participants with histories of abuse, 73% experienced one type of abuse, 20% experienced two types, and 7% had insufficient data to determine the number of abuse types experienced. With respect to the perpetrator, 51% of participants who experienced abusive acts of commission were abused by a maternal perpetrator, 49% by a paternal perpetrator, and 32% by a non-parental perpetrator (not mutually exclusive).

In order to separate participants who had not experienced abuse and/or neglect from those with missing data, the abuse and neglect variables were coded as missing if (a) the participant was not coded as having been abused based on the available information and (a) the participant was missing two or more full assessments within any given developmental period. Within the current sample, 15 participants were classified as having missing information related to abuse. The remaining 94 individuals comprised the non- abused group; the number of missing assessments for this group did not differ from the group of individuals who were classified as having experienced abuse (*t* [108.61] = −1.32, *p* = .19).

As noted above, although the MLSRA has sufficient prospective physical neglect data these data were not included in the present analysis given that the retrospective coding system does not code for physical neglect as it focuses on acts of commission (i.e., physical and sexual abuse) rather than omission (i.e., physical neglect). However, the present analyses examined prospective abuse in two primary ways: (a) examining indicators of overall presence of abuse and (b) examining specific parameters of abuse. To examine abuse overall, a binary variable indicating whether physical or sexual abuse occurred any time between birth and 17.5 years of age. Additionally, more nuanced characteristics of abuse were examined, including (a) the type of abuse (i.e., physical and sexual abuse) and (b) the perpetrator of abuse (i.e., mother/mother figure, father/father figure, and non-parental figure). All parameters of abuse were coded on a dichotomous basis of whether the event occurred (e.g., abused vs. not abused by mother figure).

The prospective variables used in the present analyses were based on variables previously used in the MLSRA (e.g., Nivison et al., 2021b; Raby et al., 2017). However, as noted, the present analysis focused on physical and sexual abuse, but not neglect as it was not possible to code neglect retrospectively in the context of the AAI. Therefore, the existing prospective variables were adjusted to remove neglect. See Supplemental Table 1 for a guide to the prospective variables.

#### Retrospective abuse

Experiences of overall retrospective abuse were coded in the context of the AAI at ages 19 and 26 years. The AAI is a 20-question semi-structured interview that asks individuals to recall their experiences with their primary caregivers in childhood and the effects they believe these experiences have had on their adult personality. The AAI also inquires about experiences of loss/grief, rejection, separation, and trauma/abuse. Although the AAI was not originally developed to function as a retrospective measure of abuse experiences, Leon et al. (2004) developed a scale to assess physical and sexual abuse in the context of the AAI, which was in turn adapted from the original abuse scales in the Main and Goldwyn coding system (see also Jacobvitz et al., 2006). This coding scheme allows coders to make independent judgments based on the information provided in the AAI to determine whether abuse experiences had occurred. Although the AAI does explicitly ask participants if they have experienced abuse (in the latter half of the interview), coding judgments were not based on the participants’ response to this single question (i.e., coders did not take into account an individual’s own perception of the abuse, but rather focused on the specific details of the experiences disclosed).

AAIs were coded for experiences of physical and sexual abuse on a nine-point severity of abuse scale: physical and sexual abuse were coded separately, resulting in two nine-point severity abuse scales. Individuals who did not indicate any experiences of abuse in the AAI received a score of one. Low-scoring individuals (i.e., a score of 2–3) described occasional spankings, frequent, but not harsh spankings or comments with sexual connotations. Those scoring a middle to higher score (i.e., 4–7) described experiences of harsh physical contact (e.g., spanking) that did not reach the threshold of abuse in the original AAI coding systems, extreme threats, or experiences that the individual was told about but does not explicitly recall happening to them. Finally, individuals with a high score (i.e., 8–9) described incidents of severe physical or sexual abuse, which also meet the criteria of the original AAI coding manual for abuse (Main et al., 2003–2008). High scores included behaviors such as repeated hitting of the child in the face, physical contact that leaves a mark, severe hitting that results in the child experiencing extreme fear of the parent, and any sexual contact between the adult perpetrator and the child. A dichotomous variable was created based on the presence of abuse meeting legal criteria (consistent with Jacobvitz et al., 2006; Leon et al., 2004) for both the physical and sexual abuse scales.

Participants who received high scores (8–9) on either the physical or sexual abuse scales were placed in the abuse category consistent with Leon et al. (2004). All AAIs were coded by two doctoral students (i.e., the first and second author of the present report) who were trained by the creator of the retrospective abuse coding system (author DBJ). Coder one (author MDN) coded 100% of the 19-year and 26-year AAIs. Coder two (author CRF) coded 33% of cases for reliability. Both coders were blind to the prospective abuse data. Coders demonstrated excellent interrater reliability for both the 19-year (physical abuse: ICC = .98; sexual abuse: ICC = .94, *n* = 52) and 26-year AAIs (physical abuse: ICC = .98; sexual abuse: ICC = .95, *n* = 52). The individuals who coded the 19- and 26-year AAIs for retrospective abuse were not involved in any other coding of the MLSRA AAIs nor the coding of the prospective abuse data. For the present analytic frame (*n* = 162), 14 participants had only the 19-year AAI, 5 participants had only the 26-year AAI, and 143 participants had both the 19- and 26-year AAIs. If participants only had one AAI, data were drawn from that assessment – for participants who had both assessments, scores were averaged across the 19- and 26-year AAIs.

AAIs were coded for recalled experiences of retrospective physical and sexual abuse on a continuous 1–9 scale with 1 representing no abuse and 9 representing severe abuse. Consistent with prior work (Jacobvitz et al., 2006; Leon et al., 2004), the scales were transformed from the continuous scales into a dichotomous variable representing whether the participant retrospectively reported abuse. Individuals scoring 1–7 on the scale were coded as zero indicating that they had not recalled severe abuse, and individuals scoring an 8 or 9 were coded as a one indicating that they had recalled severe abuse. This cutoff threshold was previously established by the original scale developers (Jacobvitz et al., 2006; Leon et al., 2004). This resulted in the following dichotomous variables: “recalled physical abuse age 19,” “recalled sexual abuse age 19,” “recalled physical abuse age 26,” and “recalled sexual abuse age 26,” wherein (0 = non-abused, 1 = abused). To further aggregate abuse data, an overall abuse variable was created within each assessment (i.e., 19 and 26 years) with reports of neither physical or sexual abuse coded as a 0 (i.e., “non-abuse” group) and reports of one or both types of abuse coded as a 1 (i.e., “abuse” group). This resulted in the following dichotomous variables: “recalled abuse age 19” and “recalled abuse age 26.” Finally, the recalled abuse variables at ages 19 and 26 were averaged to create an overall “omnibus retrospective abuse” variable. If an individual had reported any abuse at either the 19- or 26-year assessment, they were scored a one. If there was no abuse reported at either assessment, they were scored a zero. For individuals with only one AAI, the omnibus variable was based on whether abuse had been recalled at that assessment only. The omnibus variable was the primary retrospective variable used in the current analyses. See Supplemental Table 2 for the retrospective variable guide.

Based on previous research interest in sensitivity analyses (i.e., Cicchetti, 2013), we also examined Aim 1 using continuous versions of the retrospective scales as well as an overall severity of abuse variable from the prospective abuse data (consistent with prior MLSRA work, e.g., Nivison et al., 2021b). The severity of prospective abuse variable was a scale of total experiences of abuse calculated by summing the number of types of abuse (i.e., physical and sexual abuse) in each developmental period (i.e., infancy, early childhood, middle childhood, and adolescence). Given that each of the subtypes was coded on a dichotomous basis for each developmental period, the total experiences of abuse scale ranged from a theoretical minimum of zero (i.e., the participant did not experience any abuse in any developmental period) to a theoretical maximum of 8 (i.e., the participant experienced both physical and sexual abuse in each developmental period). The continuous retrospective scale was based on the original coding scale prior to dichotomization. Additionally, to extend Aim 2, we examined whether the magnitude of associations of retrospective and prospective measures varied by developmental period. These analyses were based on the notion that infantile amnesia may account for the low agreement between retrospective and prospective measures (e.g., Hardt & Rutter, 2004). Finally, we examined whether the agreement was influenced by the timing of the retrospective assessment (i.e., 19- vs. 26-year assessment) to determine whether the agreement varied within young adulthood.

## Results

**Figure 1.**
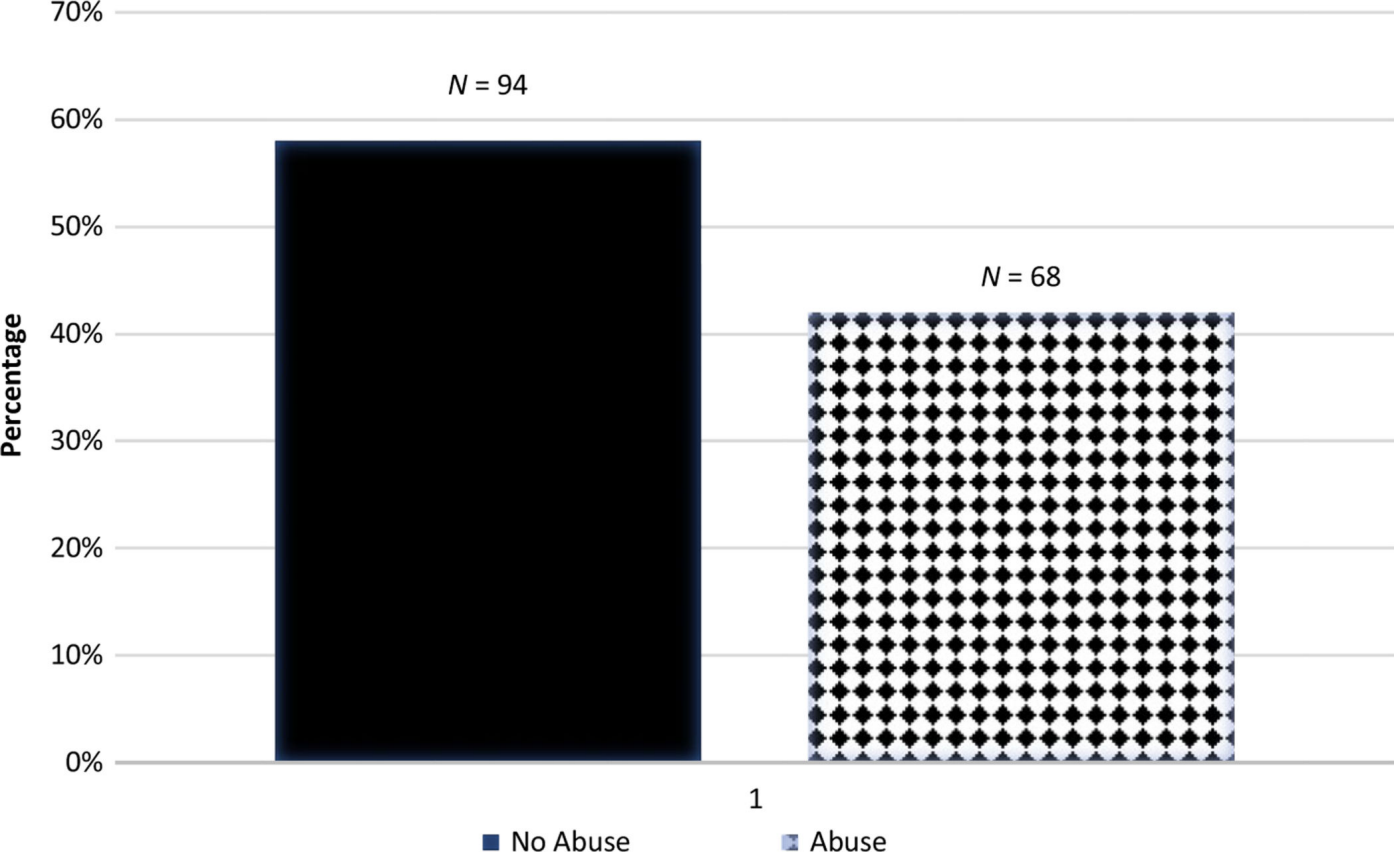
Overall abuse prospectively documented from birth to 17.5 years.

**Figure 2.**
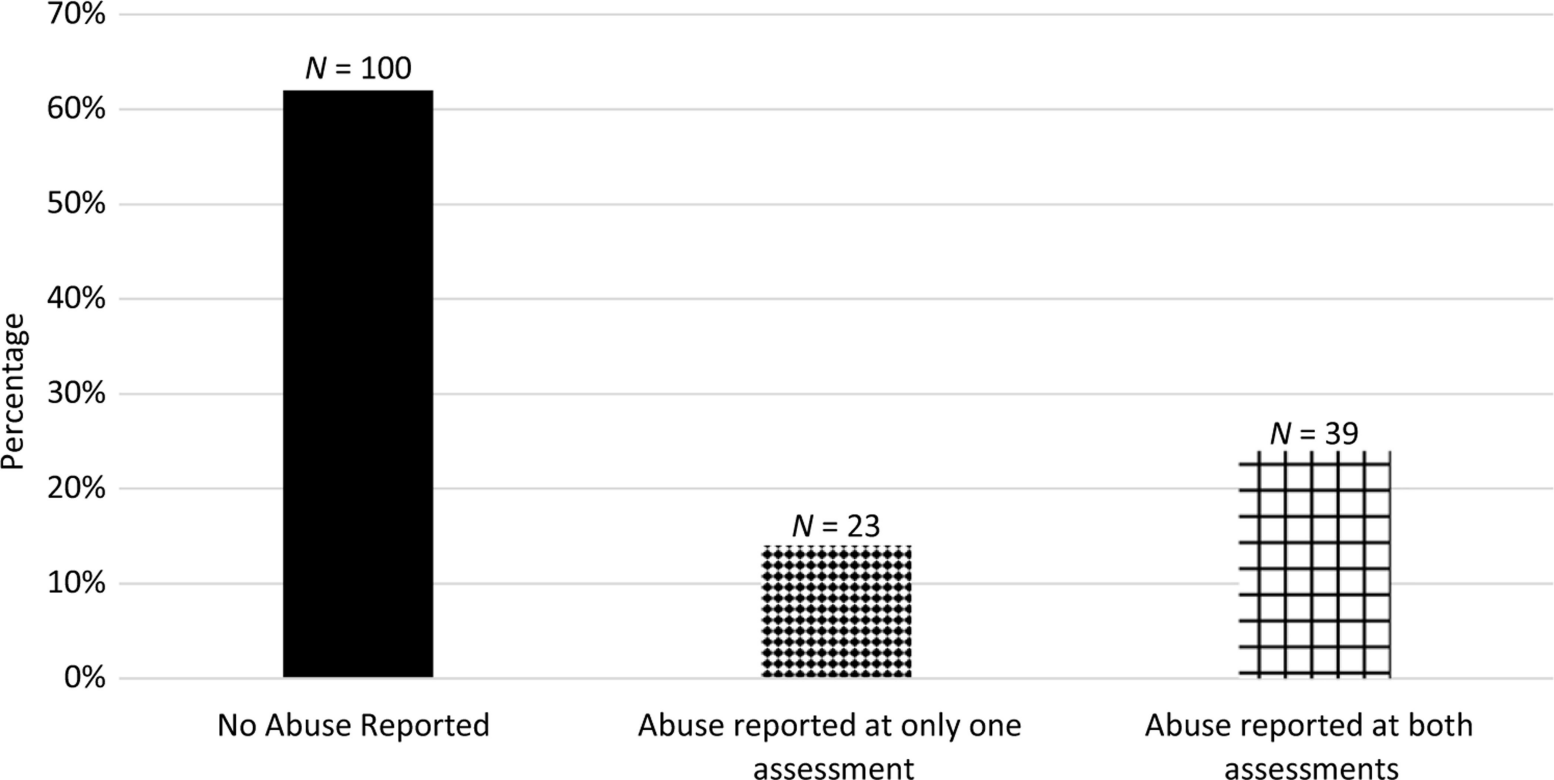
Overall abuse retrospectively reported at ages 19 and 26.

**Figure 3.**
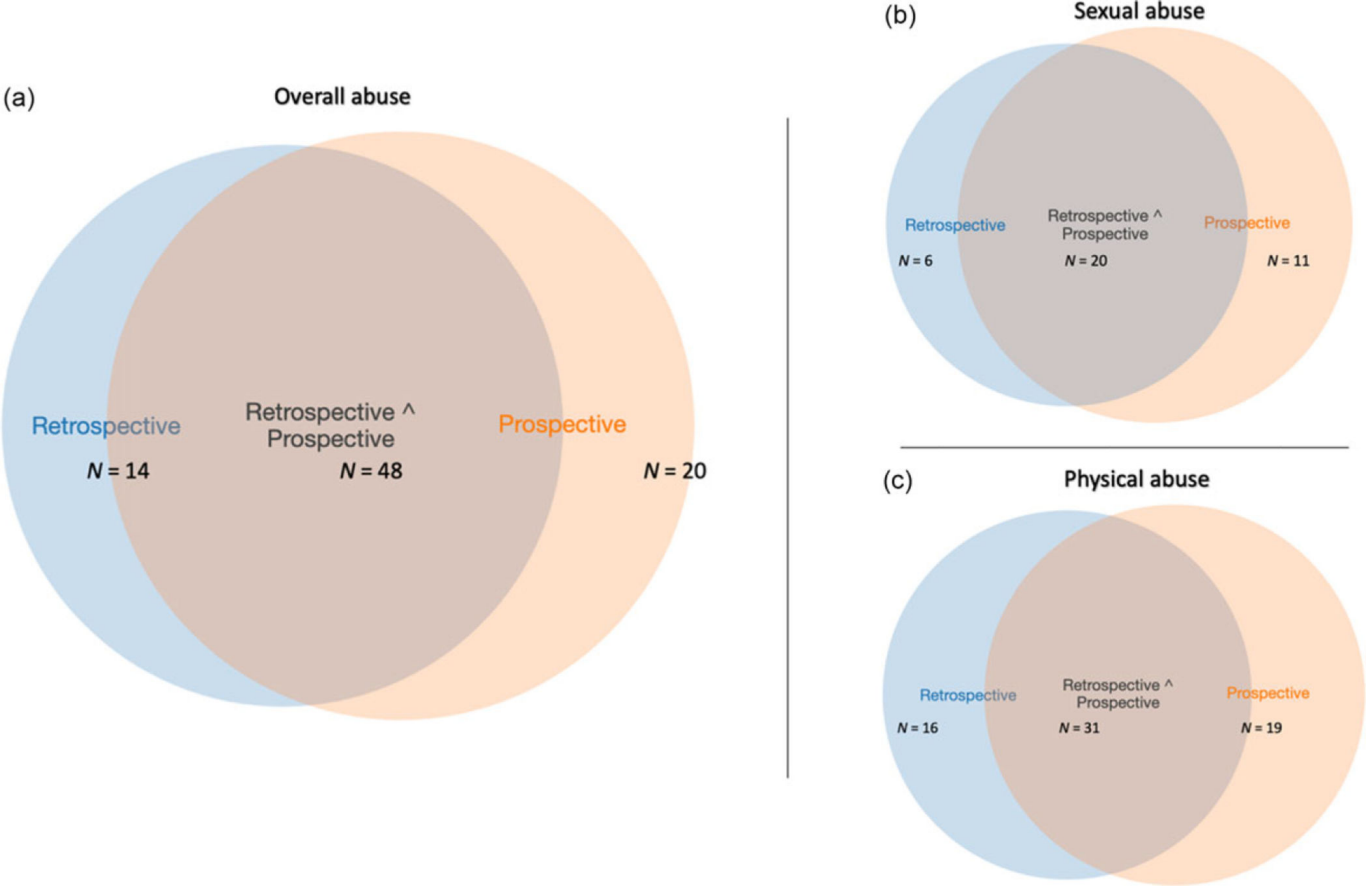
**(a)** Overall agreement between retrospective and prospective abuse. N based on individuals who were identified as having experienced physical and/or sexual abuse at either/both retrospective and prospective assessments (*n* = 82). (b) Agreement between retrospective and prospective assessments of sexual abuse. N based on individuals who were identified as having experienced sexual abuse at either/both the retrospective and prospective assessments (*n* = 37). (**c**) Agreement between retrospective and prospective assessments of physical abuse. N based on individuals who were identified as having experienced physical abuse at either/both the retrospective and prospective assessments (*n* = 66).

Descriptive statistics for all prospective and retrospective abuse variables are presented in Supplemental Tables 3 and 4, respectively. Forty-two percent of participants were prospectively identified as having experienced physical or sexual abuse anytime in the first 18 years (see Figure 1). Additionally, 38% of participants were retrospectively identified as having had at least one experience of abuse based on either the 19- or 26-year assessments (see Figure 2). The distribution of overall abuse documented prospectively, and overall abuse identified retrospectively are presented in Figures 1 and 2, respectively (the distribution of the continuous scales are presented in Supplemental Figures 1 and 2). For a breakdown of when abuse was documented (either retrospectively, prospectively, or both, see Table 1 and Figure 3).

For the following analyses, the agreement between retrospective and prospective measures of abuse was based on guidelines outlined in the interrater reliability literature (i.e., McHugh, 2012) with the important caveat that the inter-rater reliability guidelines focus on examining reliability between multiple coders for the same assessment rather than examining the reliability of two measures across the life span. In short, we adopted a priori the following approximate conventions: values between 0 and .20 were considered to have no agreement; values between .21 and .39 were considered to have minimal agreement; .40–.59 = weak agreement; .60–.79 = moderate agreement; .80–.90 = strong agreement; and above .90 = almost perfect agreement. The following Tables referenced also include the bivariate correlations as an additional metric, but the interpretation of agreement was based on the Cohen’s kappa analyses.

### To what extent does retrospectively recalled abuse in young adulthood agree with prospectively documented abuse from birth to 17.5 years?

To address Aim 1, Cohen’s kappa was estimated between the omnibus retrospective abuse variable and the prospective ever-abused variable. Retrospectively identified abuse and prospectively documented abuse demonstrated weak-to-moderate agreement (*κ* = .56). Results are outlined in Table 2. Sensitivity analyses revealed continuously measured retrospectively identified abuse, and the severity of abuse prospectively documented demonstrated moderate agreement (ICC = .65, see Supplemental Table 5). Sensitivity analyses also revealed that agreement was similar for the 19-year (i.e., *κ* = .46 and 26-year assessments i.e., *κ* = .51) and that convergence with prospective data was increased via compositing the retrospective data (results are outlined in Supplemental Table 6). Results focused on the continuous scales are reported in Supplemental Table 5.^1^ Further sensitivity analyses were conducted to apply a prevalence and bias adjustment to the kappa analyses. Results were consistent with our main findings and outlined in Tables 2 and 3.

### Does the agreement of retrospective and prospective measures of abuse vary by specific parameterizations of prospectively documented abuse?

Consistent with prior work in the MLSRA (e.g., Nivison et al., 2021b), prospectively documented abuse was analyzed by type and perpetrator of abuse. Sensitivity analyses also included the timing of abuse. To address Aim 2a, Cohen’s kappas were calculated between omnibus retrospective abuse and both prospective ever physically abused and ever sexually abused. The agreement was weak to moderate for both prospective physical (*κ* = .43) and sexual abuse (*κ* = 35). To further examine the agreement by abuse type, retrospective physical abuse and retrospective sexual abuse were compared directly with the prospective physical and sexual abuse scales. Retrospective and prospective physical abuse demonstrated moderate agreement (*κ* = .49). Retrospective and prospective sexual abuse also demonstrated moderate agreement (*κ* = .64). Results are outlined in Table 2. Post hoc analyses were conducted using a bootstrapping method outlined in Vanbelle and Albert (2008), which computes the variance-covariance matrix of the kappa coefficients and then tests their homogeneity using a Hotelling’s *T*^*2*^ test. If the difference between the kappa values is statistically significant (*p* < .05), then it can be concluded that the kappas statistically differ from one another. The agreement between omnibus retrospective abuse and prospective physical abuse and the agreement between omnibus retrospective abuse and prospective sexual abuse were not statistically significantly different from one another (*T*^*2*^ = .28, *p* = .60). Furthermore, the agreement did not significantly differ from one another when the physical and sexual retrospective abuse subscales were examined in relation to the prospective physical and sexual abuse subscales (*T*^*2*^ = 1.82, *p* = .18).

To address Aim 2b, Cohen’s kappas were estimated between omnibus retrospective abuse and abuse perpetrator (mother figure, father figure, or non-caregiver), documented prospectively. The agreement was weak for abuse perpetrated by father figures (*κ* = .37) and for mother figures (*κ* = .34) and weakest for non-caregivers (*κ* = .19). Results are presented in Table 3. Post hoc analyses revealed that agreement between retrospective abuse and any of the perpetrator subtypes did not significantly differ from one another (mother vs. father: *T*^*2*^ = .19 *p* = .66; mother vs. non-caregiver: *T*^*2*^ = 1.39 *p* = .24; father vs. non-caregiver: *T*^*2*^ = 2.17 *p* = .14).

Additional sensitivity analyses examined whether agreement varied by developmental period of prospectively documented abuse. This was entirely exploratory in nature and was conducted as a sensitivity analysis given that we could not construct parallel scales in the retrospective abuse data based on developmental timing. When broken down by prospective developmental period, retrospective abuse agreed minimally with abuse occurring in infancy (*κ* = .14), weakly in early childhood and adolescence (*κ* = .31, and .27, respectively), and moderately in middle childhood (*κ* = .45). Results are outlined in Supplemental Table 7. Post hoc analyses revealed that abuse that occurred in early childhood (*T*^*2*^ = 7.39 *p* < .01) and middle childhood (*T*^*2*^ = 12.60 *p* < .01) had higher agreement than abuse that occurred in infancy – abuse that occurred in adolescence did not significantly differ in agreement than abuse that occurred in infancy (*T*^*2*^ = 2.11 *p* = .15). Abuse occurring in early childhood, middle childhood, and adolescence did not significantly differ in agreement with retrospective abuse (early childhood vs. middle childhood: *T*^*2*^ = 1.08 *p* = .30; early childhood vs. adolescence: *T*^*2*^ = .88, *p* = .35; middle childhood vs. adolescence: *T*^*2*^ = 3.60 *p* = .06).

## Discussion

In the present study, agreement between prospectively documented childhood abuse and retrospectively assessed abuse (as measured by a coding system for the AAI) was notably higher (*κ* = .56) than would be expected based on previous meta-analytic evidence (*κ* = .19; Baldwin et al., 2019). Additionally, overall agreement was somewhat higher when examining the continuous scales of retrospective and prospective abuse (ICC = .65). These findings suggest that this specific coding system in the context of the AAI may provide a better context to evaluate retrospective abuse than existing retrospective methods. Perhaps most significantly, the present coding system did not require individuals to self-identify as having been abused as most self-report, retrospective measures do (e.g., ACEs). Instead, interview coders made decisions using the AAI coding scheme to determine whether the experiences described qualified as abusive.

The present analyses focused on the average of retrospective abuse described at the 19- and 26-year assessments. However, sensitivity analyses revealed that results did not change materially when the 19- and 26-year AAIs were examined separately. This suggests one assessment in young adulthood may be sufficient to produce a retrospective measure of abuse that converges non-trivially with prospective evidence. Additionally, we found that agreement between retrospective and prospective measures somewhat varied by specific abuse parameters. Agreement of retrospective and prospective measures was slightly higher for sexual abuse (*κ* = .64) than physical abuse (*κ* = .49). Exploratory analyses examining perpetrator and developmental timing resulted in slight variations in agreement. Agreement was higher for father figures (*κ* = .37) and mother figures (*κ* = .34) and weaker for abuse perpetrated by non-caregivers (*κ* = 19). Although examining agreement by perpetrator was exploratory given that the AAI focus on primary caregiving relationships, this emphasis of the AAI (on experiences with primary caregivers generally) may explain the lower agreement for non-caregivers. Finally, sensitivity analyses revealed that agreement was weakest for abuse occurring in infancy (*κ* = .14), which is consistent with the effects of infantile amnesia on retrospective reporting of abuse (Hardt & Rutter, 2004). That said, we advise caution in interpreting this finding given that we did not have a corresponding retrospective developmental timing scale and could not directly test the claims of infantile amnesia.

In the current study, the interview-based methodology of the AAI provided a context for individuals to report potentially abusive experiences, sometimes without an awareness of the abusive nature of the experience reported. Although the participants were asked directly about experiences of abuse, the question follows spontaneously produced narratives regarding their relationships with caregivers. Thus, it is possible that the findings in the present study were stronger than previous meta-analytic evidence about the agreement between retrospective and prospective assessments of abuse because the AAI coding system we used did not require individuals to self-identify as having experienced abuse. This is particularly important given biases associated with retrospective reporting (e.g., recall bias; Loftus, 1982). Furthermore, information learned after an event occurred can reshape how an individual recalls those events. For example, in the present study, one individual reported having seen their Child Protective Services (CPS) record indicating that they had experienced abuse in childhood, but they denied that the events ever occurred. Of course, it is also possible that some of the prospective evidence was not fully accurate. Importantly, it was not the intent of the present study to either discount an individual’s reported experiences or prove whether abuse occurred, only to highlight the difficulties that surround retrospective reporting when individuals are asked to self-define abuse experiences.

In that context, we observed cases wherein abuse was identified retrospectively, but not documented prospectively. One possible explanation for this is the hidden nature of abuse and the challenge of defining and measuring child abuse that occurs in childhood (Barnett et al., 1993). In the present study, it is possible that the prospective data did not always uncover abuse that occurred, and, in these cases, retrospective reports in the AAI might be the more accurate account of what happened in the families we studied. For example, a few individuals recalled abuse, but also explicitly indicated that they did not disclose the abuse in childhood because they were too frightened to tell anyone. Although having an interview-based retrospective measure does not solve the issue of under- or overreporting of abuse, it does potentially provide researchers insight into why, historically, the agreement between retrospective and prospective reports has been low.

Although this report provides promising evidence of an assessment of retrospectively recalled child abuse that has higher agreement with prospective assessments than previously observed in the maltreatment literature, we do not claim that the method we leveraged solves the full range of limitations of retrospective reporting of childhood maltreatment nor does it permit the complex study of child abuse long suggested in the literature (e.g., Widom et al., 2004). For example, the retrospective measure we used does not provide information on the timing, chronicity, or perpetrator of the abuse or other types of maltreatment beyond physical and sexual abuse (though other retrospective measures are in principle capable of providing these parameters: see the I-CAST; Runyan et al., 2009). These factors are critically important for intervention, prevention, and policy efforts. Thus, the usefulness of the coding system reported in the current study may be limited.

### Strengths and limitations

One central strength of this report includes its use of a prospective, longitudinal design that includes data on experiences of child abuse from birth to 17.5 years (assessed via multiple informants and methodologies) and retrospective assessments at two points in young adulthood. As noted, the retrospective coding system for the AAI we used did not rely on the participant to self-identify as having been abused. Moreover, we acknowledge that there are certain circumstances where an individual’s subjective recall of events is valuable. For example, Danese (2020) has emphasized the importance of participant’s own perceptions of their abuse-related experiences (regardless of whether they map onto prospective evidence of maltreatment). Ultimately, whether the subjective aspect of the retrospective recall of childhood abuse is valuable is dependent on the research question, or the clinical/practical context wherein the retrospective measure is being deployed.

The present study has other limitations as well. First, this analysis examined only physical and sexual abuse in the context of the AAI and no other types of abuse or neglect. Second, our study was also constrained by the operationalizations of the prospective data currently available on the MLSRA cohort. Third, the retrospective measures were limited to young adulthood. Further studies are needed to examine the agreement of retrospective and prospective assessments of childhood abuse across the life span. Moreover, as often is the case with longitudinal data, there was a decline in participation rates across the assessments. Therefore, it is necessary to highlight that it is possible that we may not have a fully accurate picture of abuse experiences given the attrition in our sample. Fourth, the MLSRA was designed to sample an at-risk population, and therefore, participants who were at or below the poverty line were specifically recruited. For that reason, the present sample is likely to have higher base rates of child abuse than a general population, and further studies are needed to investigate the validity of the AAI coding system we used for retrospectively identifying abuse in more normative risk samples.

Finally, the interview-based coding system used in the current study focused on whether or not abuse occurred. It does not provide the same detailed and rigorous information afforded by prospective data collection (e.g., timing, perpetrator, etc.). Although it is reasonable to try to code for such detailed information through an interview format, in a previous attempt with the MLSRA, Shaffer et al. (2008) concluded that there was not enough available information in the AAI to extract such information. Other retrospective measures also aim to code nuanced abuse parametrizations (see Runyan et al., 2009). However, such measures rely on participants to self-identify as having experienced abuse.

Despite the evidence provided that the present coding system used in the context of the AAI may provide higher agreement between retrospective and prospective assessments of abuse, the AAI is still a rather intensive interview (∼1.5 hours), which contrasts with one of the main benefits of common self-report measures (i.e., their ease and accessibility of use). That said, given the difficulty and extensive resources needed for assessing childhood abuse *prospectively* (e.g., Barnett et al., 1993), the AAI, in comparison, is much more accessible. Additionally, over 10,000 AAIs have already been administered (see Bakermans-Kranenburg & van IJzendoorn., 2009), and therefore, this coding scale could be applied to existing interviews. Moreover, the AAI has already been shown to be of use in clinical contexts (see Steele, H & Steele, M, 2006).

## Conclusion

In the present study, agreement between retrospectively assessed abuse, as measured in the AAI, and prospectively documented abuse was notably stronger (*κ* = .56, ICC = .65) than previous meta-analytic evidence (*κ* = .19; Baldwin et al., 2019). Based on the current findings, the AAI and the related abuse coding scheme (i.e., Jacobvitz et al., 2006; Leon et al., 2004) may provide a more valid assessment of childhood experiences of physical and sexual abuse compared to existing, retrospective measures. Nonetheless, we acknowledge that additional studies are needed to test the validity of this scale for the AAI. In addition, although this report provides promising evidence of a more valid assessment of retrospectively recalled abuse, it is not enough to solve the inherent issues related with retrospective reporting. As such, for now, prospective, longitudinal evidence necessarily remains the “gold standard” when investigating the etiology and consequences of childhood experiences of abuse.

## Supplementary Material

1

## Figures and Tables

**Table 1.** Frequencies of the reports of abuse: overall, physical, and sexual abuse

Abuse documented only prospectively	Abuse only reported at the retrospective assessment	Abuse both documented prospectively and reported retrospectively	No reported abuse at either prospective or retrospective assessment
Overall abuse (*n* = 162)			
20	14	48	80
Physical abuse (*n* = 162)			
19	16	31	96
Sexual abuse (*n* = 157)			
11	6	20	120

*Note.* For sexual abuse: one retrospective report was refused; five prospective reports did not have enough information to code for SA.

**Table 2.** Comparison of retrospective abuse and type of prospective abuse

Comparison groups	Cohen’s kappa	PABAK	Pearson’s correlation	*N*
Omnibus retrospective abuse and prospective ever abused	.56	.58	.57*	162
Omnibus retrospective abuse and prospective physical abuse	.43	.48	.44*	162
Omnibus retrospective abuse and prospective sexual abuse	.35	.45	.38*	157
Retrospective physical abuse and prospective physical abuse	.49	.57	.49*	162
Retrospective sexual abuse and prospective sexual abuse	.64	.78	.64*	157

*Note.* PABAK = prevalence-adjusted bias-adjusted kappa statistic.

* *p* < .05.

**Table 3.** Comparison of retrospective abuse and perpetrator of prospective abuse

Comparison groups	Cohen’s kappa	PABAK	Pearson’s correlation	*N*
Omnibus retrospective abuse and prospective abuse by mother figure	.34	.43	.36*	161
Omnibus retrospective abuse and prospective abuse by father figure	.37	.46	.40*	160
Omnibus retrospective abuse and prospective abuse by non-caregiver	.19	.35	.23*	156

*Note.* PABAK = prevalence-adjusted bias-adjusted kappa statistic.

* *p* < .05.

## Footnotes

1 The scope of the present project was to examine the extent to which physical and sexual abuse can be accurately recalled in a retrospective assessment and therefore, individuals who were neglected (prospectively documented), but not physically or sexually abused fell into the “non-abused” group when these analyses were run. Although neglect is not specifically abuse, but is a form of maltreatment, it is possible that agreement may have varied by operationalizing neglected individuals in the “non-abuse” category. However, because the retrospective assessment did not measure neglect, we could not compare the neglect group on their own. Therefore, to fully make accurate conclusions about the extent to which physical and sexual abuse can be retrospectively recalled, sensitivity analyses were conducted excluding participants with prospectively documented cases of neglect only (i.e., did not experience physical or sexual abuse). In the current sample, 15 participants had experienced only neglect, but not physical or sexual abuse and were excluded in these sensitivity analyses. We found that results did not materially change when these 15 participants were excluded from analyses (*κ* = .56).
