# Supplementary material for: Agreement between retrospective and prospective assessments of childhood abuse revisited
Source: Dev Psychopathol. Author manuscript; Available in PMC 2025 Jul 8. (PMC11615158; doi:10.1017/S0954579424001032)
Supplement: 1 [file NIHMS1990846-supplement-1.docx]

**Supplementary Materials**

| **Supplemental Table 1.** Prospective analytical variable guide | | |
| --- | --- | --- |
| **Based on dichotomous data** | **Theoretical values** | **Label** |
| Ever abused (prospective) | 0 or 1 | Ever physically or sexually abused from 0-17.5 years, documented prospectively |
| Ever physically abused (prospective) | 0 or 1 | Ever physically abused from 0-17.5 years, documented prospectively |
| Ever sexually abused (prospective) | 0 or 1 | Ever sexually abused from 0-17.5 years, documented prospectively |
| Ever abused by mother figure (prospective) | 0 or 1 | Ever abused by mother figure from 0-17.5 years, documented prospectively |
| Ever abused by father figure (prospective) | 0 or 1 | Ever abused by father figure from 0-17.5 years, documented prospectively |
| Ever abused by non-caregiver (prospective) | 0 or 1 | Ever abused by non-caregiver from 0-17.5 years, documented prospectively |
| Ever abuse in infancy (prospective) | 0 or 1 | Ever abused in infancy, documented prospectively |
| Ever abused in early childhood (prospective) | 0 or 1 | Ever abused in early childhood, documented prospectively |
| Ever abused in middle childhood (prospective) | 0 or 1 | Ever abused in middle childhood, documented prospectively |
| Ever abused in adolescence (prospective) | 0 or 1 | Ever abused in adolescence, documented prospectively |
| **Severity of abuse (sensitivity analyses)** | **Theoretical values** | **Label** |
| Severity of abuse (prospective) | 0-8 | Sum of total experiences of physical and sexual abuse across all developmental periods 0-17.5 years, documented prospectively |

| **Supplemental Table 2.** Retrospective analytic variable guide | | |
| --- | --- | --- |
| **Based on dichotomous data** | **Theoretical values** | **Label** |
| Omnibus retrospective abuse | 0 or 1 | Any recalled abuse at either age 19 or 26 assessments |
| Recalled abuse age 19 | 0 or 1 | Any recalled abuse at the age 19 assessment |
| Recalled abuse age 26 | 0 or 1 | Any recalled abuse at the age 26 assessment |
| Recalled physical abuse age 19 | 0 or 1 | Any recalled physical abuse at the age 19 assessment |
| Recalled sexual abuse age 19 | 0 or 1 | Any recalled sexual abuse at the age 19 assessment |
| Recalled physical abuse age 26 | 0 or 1 | Any recalled physical abuse at the age 26 assessment |
| Recalled sexual abuse age 26 | 0 or 1 | Any recalled sexual abuse at the age 26 assessment |
| **Based on continuous data (sensitivity analyses)** | **Theoretical values** | **Label** |
| Continuous omnibus retrospective abuse | 0-8 | Any recalled abuse at either age 19 or 26 assessments |
| Continuous recalled abuse age 19 | 0-8 | Any recalled abuse at the age 19 assessment |
| Continuous recalled abuse age 26 | 0-8 | Any recalled abuse at the age 26 assessment |
| Continuous age 19 physical abuse | 0-8 | Any recalled physical abuse at the age 19 assessment |
| Continuous age 19 sexual abuse | 0-8 | Any recalled sexual abuse at the age 19 assessment |
| Continuous age 26 physical abuse | 0-8 | Any recalled physical abuse at the age 26 assessment |
| Continuous age 26 sexual abuse | 0-8 | Any recalled sexual abuse at the age 26 assessment |
| *Note.* The omnibus retrospective abuse variable reflects the average of the 19 and 26-year recalled abuse—values at the original assessment were either a 0 for no reported abuse, or 1 for reported abuse. Values of the omnibus (average) retrospective abuse are as follows: 0 = no reported abuse at either AAI assessment; .5 = abuse reported at only one assessment of the AAI; 1 = abuse reported at both the 19 and 26-year AAIs. | | |

| **Supplemental Table 3. Descriptive statistics for prospective analytic variables** | | | | | |  |  |
| --- | --- | --- | --- | --- | --- | --- | --- |
| **Based on dichotomous data** | | ***N*** | **Min** | **Max** | ***%* Abuse** | ***M*** | **SD** |
| Ever abused (prospective) | | 162 | 0 | 1 | 42% | - | - |
| Ever physically abused (prospective) | | 162 | 0 | 1 | 31% | - | - |
| Ever sexually abused (prospective) | | 157 | 0 | 1 | 19% | - | - |
| Ever abused by mother figure (prospective) | | 161 | 0 | 1 | 22% | - | - |
| Ever abused by father figure (prospective) | | 160 | 0 | 1 | 21% | - | - |
| Ever abused by non-caregiver (prospective) | | 156 | 0 | 1 | 14% | - | - |
| Ever abuse in infancy (prospective) | | 158 | 0 | 1 | 6% | - | - |
| Ever abused in early childhood (prospective) | | 160 | 0 | 1 | 19% | - | - |
| Ever abused in middle childhood (prospective) | | 162 | 0 | 1 | 27% | - | - |
| Ever abused in adolescence (prospective) | | 161 | 0 | 1 | 12% | - | - |
| **Severity of abuse (sensitivity analyses)** | | ***N*** | **Min** | **Max** | **% Abuse** | ***M*** | **SD** |
| Severity of abuse (prospective) | | 157 | 0 | 5 | - | 0.69 | 1.07 |
|  | *Note.* Min = minimum observed scale value, Max = maximum observed scale value.  % Abuse reflects the percentage of the population who experienced abuse (given dichotomous data). | | | | | | |

| **Supplemental Table 4. Descriptive statistics for retrospective analytic variables** | | | | | | |  |
| --- | --- | --- | --- | --- | --- | --- | --- |
| **Based on dichotomous data** | | ***N*** | **Min** | **Max** | **% Abuse** | ***M*** | **SD** |
| Omnibus retrospective abuse | | 162 | 0 | 1 | 38% | - | - |
| Recalled abuse age 19 | | 153 | 0 | 1 | 28% | - | - |
| Recalled abuse age 26 | | 146 | 0 | 1 | 36% | - | - |
| Recalled physical abuse age 19 | | 152 | 0 | 1 | 20% | - | - |
| Recalled sexual abuse age 19 | | 152 | 0 | 1 | 10% | - | - |
| Recalled physical abuse age 26 | | 146 | 0 | 1 | 29% | - | - |
| Recalled sexual abuse age 26 | | 146 | 0 | 1 | 14% | - | - |
| **Based on continuous data (sensitivity analyses)** | | ***N*** | **Min** | **Max** | **% Abuse** | ***M*** | **SD** |
| Continuous omnibus retrospective abuse | | 162 | 0 | 8 | - | 2.25 | 2.12 |
| Continuous recalled abuse age 19 | | 153 | 0 | 8 | - | 2.05 | 2.21 |
| Continuous recalled abuse age 26 | | 146 | 0 | 8 | - | 2.49 | 2.38 |
| Continuous age 19 physical abuse | | 152 | 0 | 8 | - | 3.08 | 3.07 |
| Continuous age 19 sexual abuse | | 152 | 0 | 8 | - | 0.95 | 2.48 |
| Continuous age 26 physical abuse | | 146 | 0 | 8 | - | 3.51 | 3.28 |
| Continuous age 26 sexual abuse | | 146 | 0 | 8 | - | 1.48 | 2.89 |
|  | *Note.* Min = minimum observed scale value, Max = maximum observed scale value.  % Abuse reflects the percentage of the population who experienced abuse (given dichotomous data). | | | | | | |

| **Supplemental Table 5.** Comparison of overall continuous retrospective and prospective ever abused | | | |
| --- | --- | --- | --- |
| **Comparison groups** | **Intraclass Correlation** | **Pearson’s Correlation** | ***N*** |
| 1. Continuous retrospective abuse & severity of prospective abuse | .65 | .60* | 157 |
| 1. Continuous age 19 retrospective abuse & severity of prospective abuse | .61 | .57* | 148 |
| 1. Continuous age 26 retrospective abuse & severity of prospective abuse | .56 | .53* | 141 |
| *Note.*  **p* < .05 | | | |

|  | **Supplemental Table 6.** Comparison of overall dichotomous retrospective by assessment and prospective ever abused | | | | |
| --- | --- | --- | --- | --- | --- |
| **Comparison groups** | | **Cohen’s Kappa** | **PABAK** | **Pearson’s Correlation** | ***N*** |
| Age 19 retrospective abuse & prospective ever abused | | .46 | .50 | .49* | 153 |
| Age 26 retrospective abuse & prospective ever abused | | .51 | .53 | .52* | 146 |
|  | *Note.* PABAK = Prevalence-adjusted bias-adjusted kappa statistic  **p* < .05 | | | | |

|  | **Supplemental Table 7.** Comparison of retrospective abuse and developmental period of prospective abuse | | | | |
| --- | --- | --- | --- | --- | --- |
| **Comparison groups** | | **Cohen’s Kappa** | **PABAK** | **Pearson’s Correlation** | ***N*** |
| Omnibus retrospective abuse & prospective abuse in infancy | | .14 | .33 | .21* | 158 |
| Omnibus retrospective abuse & prospective abuse in early childhood | | .31 | .41 | .32* | 160 |
| Omnibus retrospective abuse & prospective abuse in middle childhood | | .45 | .51 | .49* | 162 |
| Omnibus retrospective abuse & prospective abuse in adolescence | | .27 | .40 | .40* | 161 |
|  | *Note.* PABAK = Prevalence-adjusted bias-adjusted kappa statistic  **p* < .05 | | | | |

**Supplemental Figure 1**

*Note.* This variable is the sum of the number of types of abuse (i.e., physical and sexual abuse) in each developmental period (i.e., infancy, early childhood, middle childhood, and adolescence). 0 reflects no instances of abuse in any developmental period.

**Supplemental Figure 2**

*Note.* The above variable is the average of the 19 and 26-year physical and sexual abuse scales that were coded on a 0-8 basis. 0 indicates no recalled experiences of any type of abuse during either assessment.

**
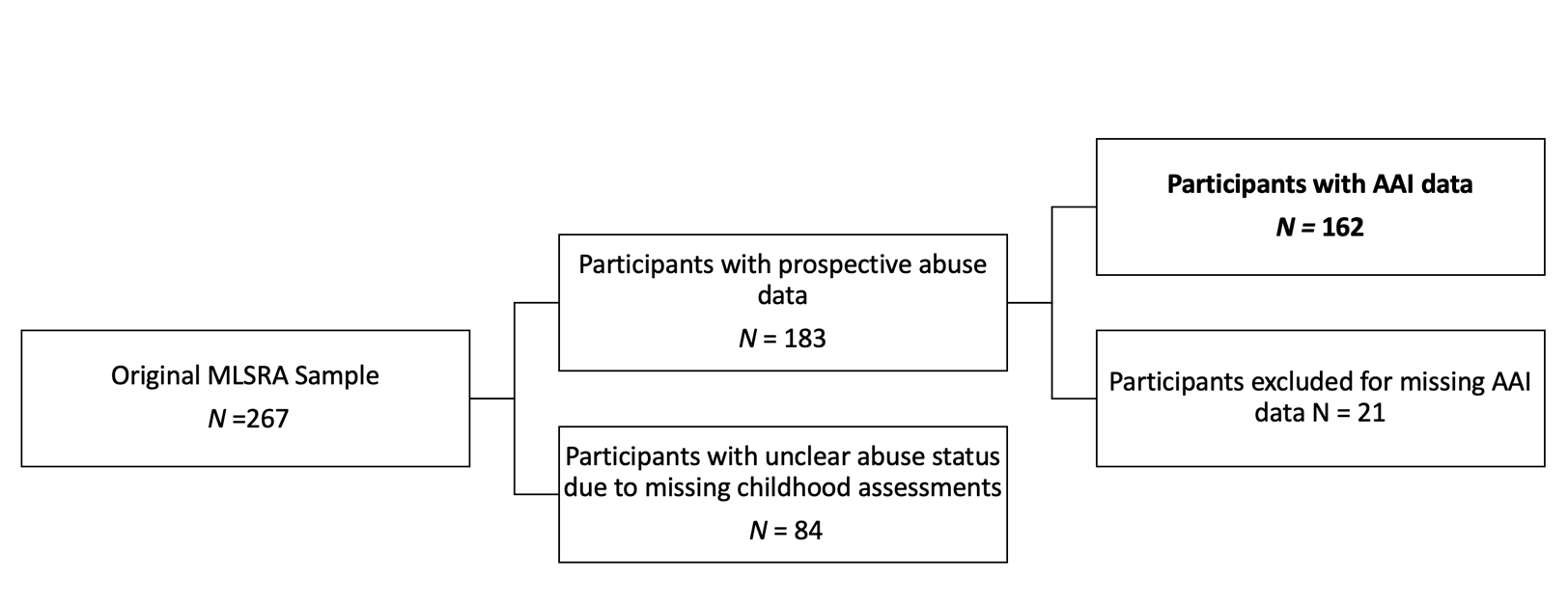
Supplemental Figure 3.** Present Subsample Selection Flowchart

**Note.** Bolded box indicates the final subsample (*N* = 162)
